# Medication adherence and quality of life among the elderly with diabetic retinopathy^1^

**DOI:** 10.1590/0104-1169.3477.2494

**Published:** 2014

**Authors:** Fernanda Freire Jannuzzi, Fernanda Aparecida Cintra, Roberta Cunha Matheus Rodrigues, Thaís Moreira São-João, Maria Cecília Bueno Jayme Gallani

**Affiliations:** 2 Doctoral student, Faculdade de Enfermagem, Universidade Estadual de Campinas, Campinas, SP, Brazil; 3 PhD, Associate Professor, Faculdade de Enfermagem, Universidade Estadual de Campinas, Campinas, SP, Brazil; 4 Post-doctoral fellow, Faculdade de Enfermagem, Universidade Estadual de Campinas, Campinas, SP, Brazil; 5 PhD, Associate Professor, Faculdade de Enfermagem, Universidade Estadual de Campinas, Campinas, SP, Brazil. Full Professor, Faculté des sciences infirmières, Université Laval, Québec, Canada

**Keywords:** Medication Adherence, Diabetic Retinopathy, Quality of Life, Health of the Elderly, Vision, Ocular

## Abstract

**OBJECTIVE:**

to investigate the factors related to medication adherence and its relation to Health- Related Quality of Life (HRQoL) in elderly people with diabetic retinopathy.

**METHOD:**

one hundred (n=100) elderly outpatients with diabetic retinopathy taking antihypertensives and/or oral antidiabetics/insulin were interviewed. Adherence was evaluated by the adherence proportion and its association with the care taken in administrating medications and by the Morisky Scale. The National Eye Institute Visual Functioning Questionnaire (NEI VFQ-25) was used to evaluate HRQoL.

**RESULTS:**

most (58%) reported the use of 80% or more of the prescribed dose and care in utilizing the medication. The item "stopping the drug when experiencing an adverse event", from the Morisky Scale, explained 12.8% and 13.5% of the variability of adherence proportion to antihypertensives and oral antidiabetics/insulin, respectively.

**CONCLUSION:**

there was better HRQoL in the Color Vision, Driving and Social Functioning domains of the NEI VFQ-25. Individuals with lower scores on the NEI VFQ-25 and higher scores on the Morisky Scale presented greater chance to be nonadherent to the pharmacological treatment of diabetes and hypertension.

## Introduction

The visual acuity decrease in the elderly contributes expressively to accentuate their dependence, by the changes related to social and psychological aspects, the gradual loss of autonomy, self-care and quality of life^(^ 1 ^)^. Diabetic retinopathy (DR) constitutes one of the most incapacitating microangiopathic complications in older patients with diabetes mellitus (DM)^(^ 2 ^)^ and it is divided into two phases: non-proliferative and proliferative. Non-proliferative DR is characterized by intra-retinal alterations associated with the increase in capillary permeability and, occasionally, to vascular occlusion^(^ 3 ^)^. In the progression of non-proliferative DR, the formation of new veins can be observed at the vitreous interface of the retina, constituting the proliferative DR^(^ 3 ^)^.

The risk factors for DR are basically hyperglycemia and hypertension^(^ 4 ^)^, pointing to the importance of the regular use of medications to control glycemia and pressure levels, in order to prevent the manifestation of the disease and/or its evolution. This is an important issue among the elderly population that has demonstrated a tendency to nonadherence^(^ 5 ^)^. Moreover among those affected by DR, the failure to adhere to drug therapy results in the inadequate control of the glycemia and hypertension, in the progression of retinal complications, and in the worsening of the visual acuity that, in turn, compromises the quality of life of these individuals.

The health-related quality of life (HRQoL) has been thoroughly studied in the elderly population. Epidemiological and clinical studies have analyzed the perception of health status and HRQoL among the elderly, as well as the impact of their disease and its respective treatment in HRQoL^(^ 6 ^)^. However, only few researches report the evaluation of vision-related quality of life^(^ 7 ^)^.

Therefore, assuming that medication adherence in the elderly with DR could be influenced by sociodemographic conditions, low visual acuity and HRQoL, this study intended to analyze the correlation/association between medication adherence and the sociodemographic/clinical variables and HRQoL of the elderly with DR. More specifically, the study evaluates: 1. the elderlys' adherence to the specific medications (oral antidiabetics/insulin and antihypertensives); 2. vision-related quality of life of these individuals; and 3. the relation between medication adherence, sociodemographic/clinical variables and vision-related quality of life.

## Methods

### Design, Settings and Sample

This descriptive, cross-sectional, correlational study was conducted in an Ophthalmological outpatient clinic of a university hospital in the interior of the state of São Paulo, Brazil. The study recruited one hundred (100) elderly individuals with medical diagnosis of diabetic retinopathy in use of antihypertensives and/or oral antidiabetics/insulin. Elderly presenting: 1. Visual acuity loss occurring secondary to multiply causes (glaucoma, congenital ocular diseases, media opacity); and 2. Ocular surgeries thirty (30) days or less before the data collection^(^ 8 ^)^ were excluded.

### Data Collection

Data was gathered from February up to December 2008 by structured individual interviews. Data regarding the ophthalmological status were obtained from the patients' medical records, right after the medical consultation, and data concerning adherence and HRQoL by interview.

### Instruments

- *Sociodemographic and clinical data:* the instrument was composed of three parts: I. Sociodemographic profile; II. Clinical characterization; III. Ophthalmological evaluation: visual acuity for distance (Snellen Optometric Chart) and for near vision (Jaeger Table) in the better-seeing eye, with optical corrections if the patients made use. The elderly were grouped according to visual acuity for distance^(^ 9 ^)^ and near vision^(^ 10 ^)^; with minor adaptations.

*- National Eye Institute Vision Functioning Questionnaire (NEI VFQ-25), Brazilian version* ^(^ 11 ^)^: questionnaire assessing the influence of visual impairment on HRQoL. The 25-item NEI VFQ comprises 12 domains: general health, general vision, ocular pain, near activities, distance activities, social functioning, mental health, role difficulties, dependency, driving, color vision and peripheral vision. Each subscale is scored so that zero (0) represents the lowest and one hundred (100) the best possible score. In the present study, the value of internal consistency, assessed by Cronbach's alpha was 0.95.

- *Brazilian Version of the Morisky Medication Adherence Scale* ^(^ 12 ^)^, composed of four questions relative to: forgetfulness, carelessness, stopping the drug when feeling better or when experiencing adverse events. The answers are structured on a Likert-type scale, with four or five options to each item. The sum of the four items generates a score that varies from 4 up to 18. The lower is the score, greater is the favorability of adherence to the treatment. For individuals using both antihypertensives and oral antidiabetics/insulin, the Morisky Scale was applied separately for each group of medications.

- *Measurement of Medication Adherence:* adherence was evaluated regarding the proportion and the global evaluation of adherence.

*- *Proportion of adherence: evaluated according to four charts that comprehend: 1. Class, dose and dosage form of all prescribed drugs; Use of each of the prescribed medications: 2. In the 24 hours prior to the interview; 3. During the week prior to the interview; and 4. Over the month preceding to the interview. The purpose of Charts 2 and 3 was to facilitate obtaining more accurate answers by minimizing the bias of memory. Adherence was calculated based on the omitted doses, informed by the patient, by using the following calculation: [(prescribed doses - omitted doses) x 100 / prescribed doses]^(^ 13 ^)^. The respondents taking doses superior to those prescribed have their adherence value converted to the corresponding index, inferior to 100% (i.e. a patient taking 120% of the prescribed treatment was described as taking 80% of the dose)^(^ 14 ^)^. For those taking more than one class of medication, the final proportion of adherence was calculated by the average of the percentages of adherence of each medication. The proportion of adherence was considered as a continuous variable (taking the average of the proportion of all prescribed medications used), and as a categorical variable: *adequate dose* (for a proportion equal or superior to 80% of the prescribed dose) and *insufficient dose* (when the dose taken did not reach 80% of the prescribed).

*- *Global evaluation of adherence: aside from the proportion of adherence, its dosage form was also evaluated, that is, the number of times the medication was taken and its association with temporal markers: fasting, breakfast, lunch and dinner. Therefore, for the global evaluation of adherence, the patients were classified in two groups: I: *Adherent*: adequate dose and care; II: *Nonadherent*: inadequate dose and/or care.

The order of administration of NEI VFQ-25, Morisky Scale and Medication Adherence Identification was varied randomly to minimize order effects.

### Statistical Analysis

The *Statistical Package for Social Sciences* software (SPSS - version 15.0 for Windows) was used for the following analyses: descriptive, comparative (Chi-Squared and Fisher's Exact, Mann-Whitney, Kruskal-Wallis tests and Spearman correlation coefficient); logistic regression with stepwise criterion of variables selection. The strength of correlation coefficients (r) was classified as: little if any (values between 0 and 0.25), low (0.26 - 0.49), moderate (0.5 - 0.69), high (0.70 - 0.89) and very high correlation (0.9 - 1)^(^ 15 ^)^. Non-parametric tests were used since the distribution of the variables was not normal. The significance level adopted was 5%.

### Ethical aspects

All enrolled patients signed the Informed Consent Form. The study was approved by the local ethics committee (Document no. 777/2007).

## Results

### Sample characterization

The sample was predominantly female (62%), mean age of 69.5(7.1) years, schooling of 4.0(3.0) years, professionally inactive (64%). The majority of patients (85%) presented DM and hypertension and 25% only DM. Large portion of the sample had normal or near-normal distance vision (44%) and near vision (63%). Fifty-two individuals presented the non-proliferative DR; 46 proliferative DR and 2 experienced both. The DR diagnosis length was in average 32.7(25.6) months varying from 6 up to 180 months.

### Evaluation of Vision-Related Quality of Life (NEI VFQ-25)

The highest scores were observed in the "Color Vision", "Driving" and "Social Functioning" domains, with a tendency to better HRQoL in these domains. The lowest score was obtained in the "General Health" domain.

### Measurement of Medication Adherence

Among the sample, 85% utilized antihypertensive drugs and all utilized oral antidiabetics and/or insulin, 48% being insulin-dependent. The Morisky Scale was applied separately for the hypertensive medications and for the oral antidiabetics/insulin. The averages obtained were 5.4(1.7) and 5.1(1.6), respectively. During the month that preceded the interview, the individuals used 86.3% of the doses prescribed for hypertension and/or DM. Most of the studied group (58%) was considered adherent because they reported having utilized 80% or more of the prescribed dose and having taken the necessary care in utilizing the medications (Table 1).

**Table 1 -:** Medication adherence according to the criteria of proportion, Morisky Scale and classification in accordance with the adequacy of the dose and care in taking the medication (n=100). Campinas, SP, Brasil, 2008

Proportion of medication adherence (%)					
Medications	Total (n=100)		Diabetic individuals (n=15)		Diabetic and hypertensive individuals (n=85)
Antidiabetics	87.5 (19.5)		86.7 (13.3)		88.1 (20.1)
Antihypertensives	86.2 (21.1)		---		88.2 (19.6)
Antidiabetics and Antihypertensives	86.3 (18.0)		---		87.5 (18.1)
Morisky Scale					
Medications	Items	Total (n=100)		Diabetic individuals (n=15)	Diabetic and hypertensive individuals (n=85)
Oral antidiabetics and/or insulin	Total	5.1 (1.6)		4.6 (1.0)	5.2 (1.7)
	Item 1	1.4 (0.7)		1.3 (0.7)	1.4 (0.7)
	Item 2	1.4 (0.9)		1.1 (0.5)	1.5 (0.9)
	Item 3	1.1 (0.3)		1.1 (0.3)	1.1 (0.3)
	Item 4	1.3 (0.7)		1	1.3 (0.8)
Antihypertensives	Total	5.4 (1.7)		---	5.4 (1.7)
	Item 1	1.5 (0.8)		---	1.5 (0.8)
	Item 2	1.5 (0.9)		---	1.5 (0.9)
	Item 3	1.1 (0.4)		---	1.1 (0.4)
	Item 4	1.3 (0.7)		---	1.3 (0.7)
Classification of global adherence to medication therapy (n=100)					
Adherent					58%

The Morisky Scale, though treated by some authors as a measure of adherence, actually unites four factors that predict patient medication-taking behavior, without measuring adherence itself. Therefore, the correlation between the Morisky Scale (total score and score of each item) and the proportion of adherence was tested initially (Table 2).

**Table 2 -:** Correlations between the Morisky Scale and the proportion of medication adherence (n=100). Campinas, SP, Brasil, 2008

Morisky Scale		Proportion of Medication Adherence*							
		Antidiabetics			Antihypertensives			Both	
		r	p-value		r	p-value		r	p-value
Antihypertensives (n=85)	Total	--	--		-0.45	<0.001		-0.48	<0.001
	Item 1	--	--		-0.05	0.624		-0.09	0.409
	Item 2	--	--		-0.16	0.150		-0.19	0.078
Antihypertensives (n=85)	Item 3	--	--		-0.40	<0.001		-0.40	<0.001
	Item 4	--	--		-0.59	<0.001		-0.59	<0.001
Oral Antidiabetics /Insulin (n=100)	Total	-0.38	<0.001		--	--		-0.42	<0.001
	Item 1	-0.07	0.471		--	--		-0.01	0.910
	Item 2	-0.15	0.137		--	--		-0.202	0.044
	Item 3	-0.43	<0.001		--	--		-0.39	<0.001
	Item 4	-0.40	<0.001		--	--		-0.52	<0.001

r= Coefficient of Spearman's correlation. Morisky Scale: lower scores indicate better favorability for medication adherence. Items 1 and 2: range from 1 up to 5; items 3 and 4: range from 1 up to 4.

* The proportion of medication adherence presented here corresponds to the percentage of the used doses over the last month.

Negative correlations of low magnitude were observed between the total score of the Morisky Scale and the proportion of adherence of antihypertensives and antidiabetics, indicating that the more the patients revealed concordance with the items on the Morisky Scale, which lead to nonadherence, the less they correctly utilized the prescribed medications. The proportion of adherence of both antidiabetic and antihypertensive medications correlated negatively, with low to moderate magnitude, with the last two items (*stopping the drug when feeling better* or *when experiencing adverse events*).

A multivariate linear regression analysis was performed to investigate which item(s) of the Morisky Scale effectively explained the variability of the proportion of adherence (Table 3). Only the item 4 (*stopping the drug when experiencing an adverse event*) explained the variability of the proportion of adherence for both the antihypertensive (12.8%) and the oral antidiabetics/insulin drugs (13.5%).

**Table 3 -:** Analysis of multivariate linear regression of the proportion of adherence to the medications, according to the Morisky Scale (n=100). Campinas, SP, Brasil, 2008

Morisky Scale (antihypertensives) (n=85)	Proportion of adherence to the antihypertensives		
	Beta (SE)^†^	p-value	R^2^ Partial
Item 1*	-0.078 (0.119)	0.512	0.005
Item 2*	-0.163 (0.125)	0.197	0.022
Item 3*	-0.348 (0.220)	0.119	0.027
Item 4*	-0.462 (0.189)	0.017	0.128
Morisky Scale (antidiabetics/insulin) (n=100)	Proportion of adherence to the antidiabetics/insulin		
	Beta (SE)^†^	p-value	R^2^ Partial
Item 1*	-0.136 (0.119)	0.258	0.011
Item 2*	-0.154 (0.120)	0.204	0.017
Item 3*	-0.369 (0.211)	0.083	0.030
Item 4*	-0.446 (0.158)	0.006	0.135

* Variables transformed into ranks due to the absence of normal distribution.

† Beta: value of the estimate or angular coefficient (slope) in the line of regression; SE: standard error of beta; R2: coefficient of determination. R2 Total (antihypertensives): 0.180. Intercept (SE): 88.15; p< 0.001.

### Analysis of the association between adherence and the sociodemographic and clinical variables and HRQoL

With the purpose of identifying the factors possibly associated with adherence to medication therapy among the elderly, an exploratory analysis was performed, testing the correlation between adherence (analyzed as a continuous variable and the score obtained on the Morisky Scale) and the sociodemographic and clinical variables.

The correlation matrix among adherence and the sociodemographic and clinical variables demonstrated that the proportion of adherence was positively correlated with the monthly income (r=0.39; p<0.000), only for the use of the oral antidiabetics/insulin. The number of associated medications was weakly positively correlated with the item 2 (carelessness - r=0.28; p=0.005 for use of antidiabetics/insulin and r=0.27; p=0.013 for use of antihypertensives) and the total score of the Morisky Scale (r=0.23; p=0.025 for oral antidiabetics/insulin exclusively); and weakly negatively correlated with the item 3 (*stopping the drug when feeling better - *r=-0.28; p=0.01 for use of antidiabetics/insulin; r=-0.27; p=0.014 for use of antihypertensives). This suggests that the greater the number of associated medications, the greater the total score, which points to nonadherence, and the greater the concordance of the patient in being careless in the use of the medications. It also indicates that the greater number of medications in use, the less the patient stops using them when feeling better.

For the analysis of factors associated to global adherence, an analysis of logistic regression was performed, including as independent variables: visual acuity (for distance and near vision), vision-related quality of life (NEI VFQ-25) and the factors related to the nonadherence, measured by the Morisky Scale. The adherence as a dependent variable was treated as categorical: *Adherent* (adequate dose and carefulness - Group I) and *Nonadherent* (inadequate dose and/or carelessness - Group II) (Table 4).

**Table 4 -:** Analysis of univariate logistic regression for global medication adherence (n=100). Campinas, SP, Brasil, 2008

Variables	Categories	p-value	O.R*	CI 95%*
Visual acuity for distance vision	Profound low vision or blindness (ref.)	--	1.00	--
	Moderate low vision	0.399	1.62	0.53 – 4.94
	Normal	0.056	2.95	0.97 – 8.94
Visual acuity for near vision	Blindness (<J6)	--	1.00	--
	Low vision (J4, J5 and J6)	0.257	2.17	0.57 – 8.26
	Normal (J1, J2 and J3)	0.069	2.51	0.93 – 6.78
NEI VFQ-25 (categorized)	0-50 (ref.)	--	1.00	--
	50-74	0.283	1.92	0.58 – 6.32
	75-100	0.024	3.34	1.17 – 9.50
Morisky Scale Antihypertensives	≥8 points (ref.)	--	1.00	--
	6-7 points	0.220	2.70	0.55 – 13.20
	4-5 points	0.010	6.53	1.57 – 27.21
	Does not use	0.077	4.50	0.85 – 23.80
Morisky Scale Oral Antidiabetics and/or Insulin	Total score (for each 1 point)	--	1.00	--
		0.007	0.68	0.51 – 0.90
Morisky Scale Oral Antidiabetics and/or Insulin (categorized)	≥7 points (ref.)	--	1.00	--
	5-6 points	0.013	5.10	1.42 – 18.32
	4 points	0.002	6.35	1.98 – 10.37

* O.R. (Odds Ratio) for medication adherence (n= 42 Nonadherent and n= 58 Adherent). CI 95% = Confidence Interval of 95% for the risk of odds ratio. Ref.: category utilized as the reference for the analysis.

The analysis indicates that the vision-related quality of life and the Morisky Scale score (for antihypertensives and oral antidiabetics/insulin) influenced the medication adherence: individuals with lower scores on the NEI VFQ-25 and higher scores on the Morisky Scale have a greater chance of becoming nonadherent.

## Discussion

This study analyzed the associations observed between medication adherence and the sociodemographic/clinical variables and HRQoL of the elderly with DR. The observed average proportion of medication taken was above 80% for the two classes of medication, indicating adherence. However, when the evaluation of the dose and care relative to the prescription was associated to this proportion, approximately half of the individuals (42%) revealed nonadherence, characterized by the associations: adequate use of the dose and inadequate care, insufficient dose and adequate care and inadequate dose and care.

The only variable observed in relation to the medication taken was the monthly income among the patients using oral antidiabetics/insulin. This is a significant corroborated finding, considering that a large part of the medications for hypertension and DM is available to the Brazilian population free of charge at the public health services. The association between nonadherence and a worse financial situation was also reported among HIV patients^(^ 16 ^)^. Income was also a risk factor for both nonadherence and secondary hospitalization in the elderly^(^ 17 ^)^. These data reveal the importance of considering the basic necessities of the population, particularly among the elderly, in the planning of intervention dealing with the promotion of adherence to the therapy.

Regarding the correlation analysis, although the items linked to carelessness and stopping the drug when feeling better have been correlated to the proportion of adherence, this was effectively explained by the item 4, as shown on the regression analysis, which refers to the interruption of the medication when feeling worse when taking it. Therefore, attention should be given to the relevance of the patient's exact perception of "worse". In other words, it is necessary to investigate if this perception actually refers to a clinical event, such as hypotension symptoms or hypoglycemia, for example. If the investigation points to adverse events that worsen the clinical manifestations or compromise the patient's well-being, the therapeutic regimen must be reviewed. If the perception is not associated to any event that configures a clinical damage, educational interventions should be designed, aimed at self-evaluating signs and symptoms, blood pressure and glycemic levels; as well as strategies that allow improve the patient's self-efficacy in the correct handling of the prescribed drug therapy.

Concerning the association between the number of medications in use and the Morisky score, educational actions should include strategies that facilitate the elderly's comprehension of the importance of the uninterrupted use of the medications, both for obtaining as well as for maintaining the desired therapeutic effect, once they were using a greater number of medications and reported greater carelessness in taking them and stopping their use when they were feeling better.

The distinction between the Proportion of Adherence/Morisky Scale and the sociodemographic and clinical variables should be emphasized. Although the relation between the Morisky Scale and the Proportion of Adherence has been established and even though it consists of a significant finding, the two measures do not quantify the same construct.

The adherence behavior is a dynamic process, difficult to be measured, and for which it there is no standard measurement or gold standard. In the clinical practice, the Morisky Scale has shown to be effective for the identification of some of the reasons for nonadherence and, at times, it has been associated with outcomes^(^ 18 ^)^.

The studies related to adherence focus mainly on the reduction of symptoms and on the evaluation of measurement instruments. However, the relations between medication adherence and the global evaluations of well-being, which would allow comprehending and better guiding the treatment^(^ 19 ^)^, are not frequently examined. The present study provides significant contribution to this matter, by measuring the vision-related quality of life and testing it as an influencing variable on medication adherence.

In the evaluation of the NEI VFQ-25, relatively high scores were observed in all the domains of the instrument. However, it was verified that the perception of "worse" vision-related quality of life was associated with the chance of 3.34 times of nonadherence. It is interesting to note that visual acuity was not associated to adherence, which makes it possible to infer that it is not the drop in visual acuity that compromises adherence, but one's perception of how much this drop negatively interferes in his/her quality of life.

Considering that the studies on the relation between quality of life and medication adherence are still scarce in the literature, the development of future investigations is suggested, by simultaneously applying a generic measure of HRQoL and analyzing the influence of other factors possibly associated with adherence.

### Limitations of the study

One of the limitations of self-reporting adherence is the effect of social desirability, which can be more accentuated in the elderly population by the responsibility that society imposes on the elderly to care for themselves^(^ 20 ^)^. For this reason, the occurrence of overestimation in the reporting of desired behavior is possible and has been shown in a similar study^(^ 21 ^)^. However, the association of the measurements used in our study allowed the detection with greater sensibility of the problem of nonadherence among the elderly studied, ratifying the relevance of the theme in this population.

The second limitation concerns the Morisky's scale reliability level, which tends to oscillate with wide variability in the different populations studied, mainly when employed in the form of dichotomous scales (yes/no type), with registers of values (Cronbach's alpha) between 0.18 and 0.61^(^ 22 ^)^. In our study, Cronbach's alpha coefficient was 0.41, indicating reliability lower than the desirable. Even so, it should be considered that the sample presented low levels of study and the internal consistency of the scale may be damaged by the fact of being composed of only four items. Despite the low reliability, the Morisky scale is still used worldwide given its accessibility and the lack of valid and reliable questionnaires that assess adherence and the factors related to it^(^ 23 ^)^.

## Conclusion

The adherence to the antidiabetic treatment was smaller among the patients with lower monthly income and the use of a larger number of medications has been related to individual risk factors for nonadherence. One of the determinant factors for the smaller proportion of adherence was the interruption of medication by the patient's perception of adverse events. The elderly's perception of how much the drop in visual acuity interferes negatively in his HRQoL has demonstrated to compromise adherence. These findings provide for nurses evidence of the need for developing and evaluating new strategies to reduce the risk of nonadherence among the elderly with DR.

## References

[1] Daien V, Peres K, Villain M, Colvez A, Carriere I, Delcourt C (2013). Visual acuity thresholds associated with activity
limitations in the elderly: the POLA study. Acta Ophthalmol.

[2] Yu Y, Feng L, Shao Y, Tu P, Wu HP, Ding X (2013). Quality of life and emotional change for middle-aged and
elderly patients with diabetic retinopathy. Int J Ophthalmol.

[3] SBD. Sociedade Brasileira de Diabetes (2014). Diretrizes da Sociedade Brasileira de Diabetes:
2013-2014.

[4] Nittala MG, Keane PA, Zhang K, Sadda SR (2014). Risk factors for proliferative diabetic retinopathy in a
latino American population. Retina.

[5] Gellad WF, Grenard JL, Marcum ZA (2011). A systematic review of barriers to medication adherence
in the elderly: looking beyond cost and regimen complexity. Am J Geriatr Pharmacother.

[6] Netuveli G, Blane D (2008). Quality of life in older ages. Br Med Bull.

[7] Källstrand-Eriksson J, Baigi A, Buer N, Hildingh C (2013). Perceived vision-related quality of life and risk of
falling among community living elderly people. Scand J Caring Sci.

[8] Cintra FA, Guariento ME, Miyazaki L (2010). Adesão Medicamentosa em idosos em seguimento
ambulatorial. Ciênc Saúde Coletiva.

[9] Centers of Diseases, Functioning & Prevention (CDC). National Center
for Health Statistics (NCHS) Classifications of Diseases, Functioning, and Disability
[online]. ICD-10-CM PDF Format. FY15_Index. ICD-10-CM Tabular list of diseases and
injuries (FY15).

[10] Lim DH, Han JC, Kim MH, Chung ES, Chung TY (2013). Factors affecting near vision after monofocal
intraocular lens implantation. J Refract Surg.

[11] Fonseca ICM (2006). Adaptação cultural do "National Eye Institute Visual
Functioning Questionnaire" (NEI VFQ-25) para idosos brasileiros com baixa visão
[dissertação de mestrado].

[12] Ferreira MCS, Gallani MCBJ (2006). Adaptação transcultural do instrumento de Morisky de
adesão à medicação para pacientes com insuficiência cardíaca. Rev Soc Cardiol Estado de São Paulo.

[13] Ventura-Cerdá JM, Mínguez-Gallego C, Fernández-Vellalba EM, Alós-Almiñana M, Andrés-Soler J (2006). Escala simplificada para detectar problemas de
adherencia (ESPA) al tratamiento antirretroviral. Farmacia Hosp.

[14] Delgado AB, Lima ML (2001). Contributo para a validação concorrente de uma medida de
adesão aos tratamentos. Psic., Saúde Doenças.

[15] Munro BH (2005). Statistical methods for health care research.

[16] Carballo E, Cadarso-Suárez C, Carrera I, Fraga J, la Fuente J, Ocampo A (2004). Assessing relationships between health-related quality
of life and adherence to antiretroviral therapy. Qual Life Res.

[17] Col N, Fanale JE, Kronholm P (1990). The role of medication noncompliance and adverse drug
reactions in hospitalizations on the elderly. Arch Intern Med.

[18] Krapek K, King K, Warren SS, George KG, Caputo DA, Mihelich K (2004). Medication adherence and associated hemoglobin A1c in
type 2 diabetes. Ann Pharmacother.

[19] Hommel KA, Davis CM, Baldassano RN (2008). Medication adherence and quality of life in pediatric
inflammatory bowel disease. J Pediatr Psychol.

[20] Cintra FA, Sawaia BB (2000). A significação do glaucoma e a mediação dos significados
de velhice na perspectiva Vygotskiana: subsídios para a educação à
saúde. Rev Esc Enferm USP.

[21] Lourenço LB, Rodrigues RC, Ciol MA, São-João TM, Cornélio ME, Dantas RA (2014). A randomized controlled trial of the effectiveness of
planning strategies in the adherence to medication for coronary artery
disease. J Adv Nurs.

[22] George J, Shalansky SJ (2007). Predictors of refill non-adherence in patients with
heart failure. Br J Clin Pharmacol.

[23] Almeida ED, Rodrigues LC, Vieira JL (2014). Estimates of adherence to treatment of vivax
malaria. Malar J.

